# Assessment of Knowledge and Awareness Levels of Lipoma and Simple Surgical Excision Among Adults in Makkah Region, Saudi Arabia

**DOI:** 10.7759/cureus.59727

**Published:** 2024-05-06

**Authors:** Medhat Taha, Ghada E Alamri, Hanan M Almuyidi, Laila O Alnashri, Mona O Almarhabi, Reham M Alessi, Sarah T Alzubaidi, Abdullah E Alamri

**Affiliations:** 1 Department of Anatomy, Umm Al-Qura University, Al-Qunfudhah, SAU; 2 Department of Medicine, College of Medicine, Umm Al-Qura University, Al-Qunfudhah, SAU; 3 General Surgery, South Qunfudhah General Hospital, Al-Qunfudhah, SAU

**Keywords:** makkah, surgical excision, risk factors, knowledge, lipoma

## Abstract

Background

Lipoma is a soft tissue tumor primarily composed of fat cells. These slow-growing, painless, subcutaneous nodules can occur in any place in the body where fat is present. Our study aims to assess the awareness, knowledge, and attitudes of Makkah region inhabitants regarding lipomas and the surgical excision method.

Methodology

This study used a cross-sectional methodology to evaluate the general public’s knowledge regarding lipomas and the surgical excision method using a self-administered questionnaire in the Makkah region from January to April 2024.

Results

A total of 367 participants were included, with the majority (56.10%) aged between 18 and 29 years. The survey revealed that 48.50% had heard about lipomas, 42.80% lacked any knowledge about them, and 26.70% acquired their information via social media. Furthermore, 31.60% believed it to affect both genders equally, 46.60% admitted uncertainty, 20.40% correctly identified that lipomas can occur at any age, and 39.80% were uncertain. Overall, 57.20% correctly identified lipomas as benign tumors composed of fat cells. Opinions diverged on whether lipomas cause pain, with 46.90% being uncertain. Moreover, 25.90% of respondents thought that surgery was the sole option for removing a lipoma, while 38.10% recognized the risk of lipoma recurrence after surgical removal. Overall, 85.60% reported never being diagnosed with a lipoma, while 4.10% had been diagnosed, predominantly with single lipomas 6.00%. There were significant differences in the participants’ marital status, with widowed people exhibiting the greatest awareness level, followed by single people.

Conclusions

Our study findings indicate a moderate level of awareness about lipomas among residents of the Makkah region. However, there are significant gaps in understanding various aspects of lipomas, including their characteristics, treatment options, and demographic distribution.

## Introduction

Within the realm of dermatological mysteries, lipomas are a silent phenomenon filled with curiosity and questions among both patients and healthcare professionals about their origins, growth patterns, and potential impact on the surrounding structures. These benign soft tissue tumors are defined as subcutaneous tumors with predominant adipocytic (fat cells) contents, which emerge as soft, painless, encapsulated, slow-growing nodules.

The World Health Organization classified soft tissue tumors in 2020 as benign mesenchymal tumors [1], therefore, it is essential to distinguish common benign lipomas from liposarcoma, especially the well-differentiated ones because of their similar characteristics [2].

Globally, lipomas are reported to affect 1% of the population, which means it affects 2.1 per 1,000 people annually [2]. They are more common in men than women and can occur in any age group, but often occur between the fourth and sixth decades of life.

Although lipomas commonly occurs on the trunk, they can also develop in other parts of the body where soft fatty tissue is abundant. Furthermore, lipomas do not always appear as subcutaneous tumors. A few cases have shown various types of lipomas involving deep fascia, muscles, bones, nerves, and other cutaneous and noncutaneous sites.

Multiple causative factors have been reported including metabolic triggers such as obesity, diabetes, and hyperlipidemia. Moreover, lipoma cases are frequently seen after physical trauma [3]. Additionally, some studies showed cases of patients where genetic factors likely contributed to lipoma formation, as 2-3% of patients with lipoma have multiple familial anomalies representing alterations of chromosomes 12 and 13 [4].

Lipoma can be diagnosed clinically or by other radiological imaging such as ultrasound (US), computed tomography (CT), and magnetic resonance imaging (MRI) in some atypical locations of lipoma. However, most subcutaneous lipomas do not require any further imaging studies, and after complete surgical excision, samples are sent for histopathological examination [5].

Several factors influence the decision to treat lipomas, including the size of the nodule as they commonly grow to 1-10 cm, with lipomas larger than 10 cm referred to as giant lipomas [6]. Furthermore, anatomical locations, symptoms such as pain, compression on the surrounding structures, and cosmetic reasons play a role in treatment options.

A histopathological screening was performed at King Fahd Hospital at the University in Saudi Arabia from 1983 to 1989 to review the morphological spectrum of adipose tissue tumors. The study revealed that of the 1,093 cases of adipose tissue tumors, 579 patients were diagnosed with lipomas, and only 36 were greater than 10 cm in diameter [7].

Through our comprehensive review, we found global and local studies targeting lipoma from different aspects such as case reports, epidemiology, and histological features of case series. However, no study focused on understanding the public’s knowledge and awareness regarding lipoma, specifically in regions such as Al-Qunfudhah. Therefore, there is a need to highlight the knowledge gaps to improve educational efforts and conduct future research.

Therefore, this study aims to assess the knowledge and awareness levels of lipomas and simple surgical excision by identifying the point of ignorance and misunderstanding among adults in the Makkah region. Moreover, we aim to increase awareness levels of various aspects of lipomas and improve the educational efforts to promote the public’s understanding of lipomas. Additionally, this research serves as a database for future research.

## Materials and methods

This cross-sectional study of adult Saudi citizens residing in the Makkah region in Saudi Arabia was conducted between January and April 2024. The study included individuals of both genders who were older than 18 years old. Participants who lived outside of the Makkah region and were younger than 18 years old were excluded. The minimum sample size needed was 367. An Arabic questionnaire was used to collect the data.

The questionnaire was a self-administered questionnaire developed by the authors and underwent validation to assess its accuracy. The questionnaire was disseminated via the social media of the selected sample.

The questionnaire was divided into nine sections. The first section included a study description and participant consent form. The second section included demographic data such as gender, age, marital status, education level, employment status, and residence. The third section included questions assessing the awareness, source of knowledge, and participants’ satisfaction with the available information. The fourth section included questions assessing participants’ general knowledge about lipoma. The fifth section included questions assessing the knowledge about the risk factors of lipoma. The sixth section included questions assessing the knowledge about the diagnostic approach of lipoma. The seventh section included questions assessing the knowledge about medical interventions for lipoma. The eighth section included questions assessing the barriers to not seeking medical attention. Finally, the ninth section included questions assessing participants’ personal experiences.

Statistical analysis

The statistical analysis was performed using SPSS version 26 (IBM Corp., Armonk, NY, USA). The categorical data were presented as frequencies and percentages. The Mann-Whitney and Kruskal-Wallis tests were used to determine the association between the knowledge score and the sociodemographic data to present medians, interquartile ranges, and p-values. A p-value <0.05 indicated statistical significance.

Ethical considerations

Ethical approval was obtained from the Biomedical Research Ethics Committee of Umm Al-Qura University, Al-Qunfudhah, Saudi Arabia (approval number: HAPO-02-K-012-2024-02-2074).

## Results

Sociodemographic data

The study assessed the knowledge and awareness levels of lipoma and simple surgical excision among adults in the Makkah region, Saudi Arabia. A total of 367 participants were included in the study after excluding individuals who refused to participate and those aged less than 18 years. The majority of respondents fell within the age range of 18-29 years (N = 206, 56.10%). Regarding gender distribution, the sample comprised predominantly females (N = 272, 74.10%). Regarding marital status, a considerable proportion of participants were single (N = 188, 51.20%). Educational attainment varied, with the majority holding a bachelor’s degree (N = 218, 59.40%). Employment status showed that a significant proportion were students (N = 149, 40.60%). Geographically, the highest number of participants resided in Qunfudhah (N = 129, 35.10%) (Table 1).

**Table 1:** Sociodemographic data (n = 367). Data are expressed as n and %.

Parameter	Category	N	%
Age (year)	18–29	206	56.10%
	30–39	36	9.80%
	40–49	80	21.80%
	50–59	41	11.20%
	60 or more	4	1.10%
Gender	Female	272	74.10%
	Male	95	25.90%
Marital status	Single	188	51.20%
	Married	151	41.10%
	Divorced	14	3.80%
	Widowed	9	2.50%
	Other	5	1.40%
Educational level	Primary	3	0.80%
	Middle	3	0.80%
	High	70	19.10%
	Diploma	52	14.20%
	Bachelor’s degree	218	59.40%
	Other	21	5.70%
Employment status	Student	149	40.60%
	Employed	139	37.90%
	Not employed	50	13.60%
	Retired	15	4.10%
	Other	14	3.80%
Residence	Qunfudhah	129	35.10%
	The Holy Capital	92	25.10%
	Jeddah	55	15.00%
	Rabigh	3	0.80%
	Taif	38	10.40%
	Leith	4	1.10%
	Other	46	12.50%

General knowledge and awareness about lipoma

**Figure 1:**
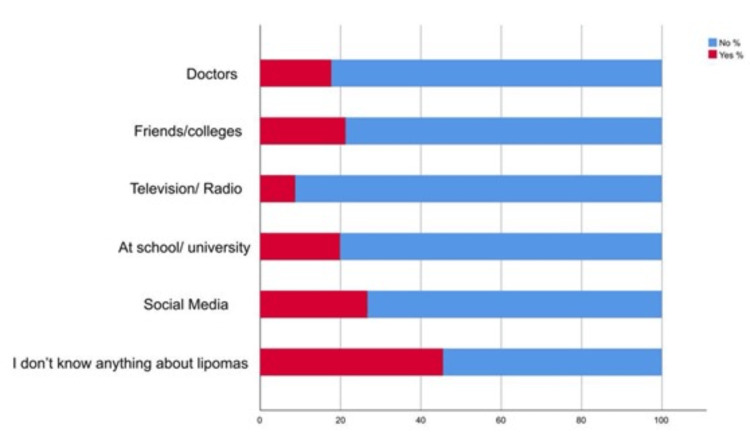
Stack bar chart showing the sources of knowledge about lipoma. Data are expressed as percentages.

As shown in Table 2, 48.50% (N = 178) of respondents had heard about lipomas, while 42.80% (N = 157) did not possess knowledge about lipomas. Regarding satisfaction with available information, responses varied, with 31.10% (N = 114) expressing the lowest satisfaction (rating 1). In terms of factual understanding, a majority (N = 210, 57.20%) correctly identified lipomas as benign tumors composed of fat cells. On the contrary, when asked about growth characteristics, responses were more divided, with 24.00% (N = 88) believing lipomas are fast-growing tumors and 52.60% (N = 193) being unsure. Similarly, opinions diverged on whether lipomas cause pain, with 46.90% (N = 172) being uncertain. A significant proportion (N = 171, 46.60%) acknowledged that lipomas may compress surrounding nerves and structures causing discomfort. Additionally, 46.30% (N = 170) were unsure if lipomas were typically hard and firm or not. Similarly, 49.60% (N = 182) were unsure if lipomas typically move freely under the skin or not. Furthermore, 190 (51.80%) participants acknowledged that lipomas can develop anywhere on the body. Finally, when asked about the understanding of the exact cause of lipomas, 64.00% (N = 235) remained uncertain about the exact etiology. As shown in Figure 1, 45.50% (N = 167) of the participants did not know anything about lipomas while 26.70% (N = 98) reported using social media as a source of knowledge about lipomas.

**Table 2:** General knowledge and awareness about lipoma (n = 367). Data are expressed as n and %.

Parameter	Category	N	%
Have you ever heard about lipoma?	No	161	43.90%
	Yes	178	48.50%
	I don’t know	28	7.60%
Do you know any information about lipoma?	No	157	42.80%
	Yes	133	36.20%
	I don’t know	77	21.00%
How satisfied are you with the available information about lipomas?	1	114	31.10%
	2	71	19.30%
	3	102	27.80%
	4	28	7.60%
	5	52	14.20%
Are lipomas benign tumors composed of fat cells?	No	15	4.10%
	Yes	210	57.20%
	I don’t know	142	38.70%
Are lipomas typically fast-growing tumors?	No	86	23.40%
	Yes	88	24.00%
	I don’t know	193	52.60%
Are lipomas commonly associated with pain?	No	104	28.30%
	Yes	91	24.80%
	I don’t know	172	46.90%
Do lipomas compress on the surrounding nerves and structures causing discomfort?	No	27	7.40%
	Yes	171	46.60%
	I don’t know	169	46.00%
Are lipomas typically hard and firm to touch?	No	87	23.70%
	Yes	110	30.00%
	I don’t know	170	46.30%
Do lipomas typically move freely under the skin?	No	29	7.90%
	Yes	156	42.50%
	I don’t know	182	49.60%
Can lipomas develop anywhere on the body?	No	18	4.90%
	Yes	190	51.80%
	I don’t know	159	43.30%
Is the exact cause of lipomas well understood?	No	93	25.30%
	Yes	39	10.60%
	I don’t know	235	64.00%

Risk factors, diagnosis, and medical interventions related to lipoma

The study assessed various aspects of knowledge and perceptions regarding lipomas and their management among participants. Regarding the demographic distribution of lipoma occurrence, 116 (31.60%) respondents believed it to affect both genders equally. A significant proportion (N = 171, 46.60%) admitted uncertainty regarding gender predilection. Regarding age distribution, 20.40% (N = 75) correctly identified that lipomas can occur at any age, while 39.80% (N = 146) were uncertain. When queried about genetic predisposition, 22.30% (N = 82) acknowledged its role, whereas 58.00% (N = 213) were uncertain. Additionally, 82 (22.30%) respondents believed that lipomas were associated with underlying health conditions and there was a link between trauma and lipoma formation. Interestingly, 148 (40.30%) respondents believed lifestyle factors could contribute to lipoma formation, and 97 (26.40%) respondents associated it with obesity. Additionally, 148 (40.30%) respondents believed there was a risk of lipomas transforming into cancerous tumors. Regarding imaging, 164 (44.70%) respondents were certain that imaging tests such as US or MRI were always necessary to diagnose lipomas, and 136 (37.10%) respondents felt treatment was usually required. Notably, 176 (48.00%) respondents recognized that larger or symptomatic lipomas may require different treatment approaches. Furthermore, 143 (39.00%) respondents acknowledged the practice of monitoring lipoma growth before recommending treatment. Regarding treatment methods, 95 (25.90%) respondents believed surgery was the sole method for removal, while 140 (38.10%) respondents recognized the risk of lipoma recurrence after surgical removal. Moreover, regarding the awareness of simple surgical excision, 181 (49.30%) respondents expressed the lowest level of awareness (rating 1), while only 9.00% (N = 33) expressed the highest level (rating 5), indicating considerable variability in awareness levels among participants (Table 3).

**Table 3:** Risk factors, diagnosis, and medical intervention regarding lipoma (n = 367). Data are expressed as n and %.

Parameter	Category	N	%
Lipomas are more common in	Males	22	6.00%
	Females	58	15.80%
	Both	116	31.60%
	I don’t know	171	46.60%
Lipomas are more common in which age group?	<20	13	3.50%
	20–40	50	13.60%
	40–60	66	18.00%
	>60	17	4.60%
	Occur at any age	75	20.40%
	I don’t know	146	39.80%
Do lipomas have a genetic predisposition in some cases?	No	72	19.60%
	Yes	82	22.30%
	I don’t know	213	58.00%
Are lipomas associated with underlying health conditions?	No	58	15.80%
	Yes	82	22.30%
	I don’t know	227	61.90%
Is there a link between trauma and lipoma formation?	No	58	15.80%
	Yes	82	22.30%
	I don’t know	227	61.90%
Can lifestyle factors contribute to the formation of lipomas?	No	28	7.60%
	Yes	148	40.30%
	I don’t know	191	52.00%
Are lipomas associated with obesity?	No	82	22.30%
	Yes	97	26.40%
	I don't know	188	51.20%
Is there a risk of lipomas transforming into cancerous tumor?	No	35	9.50%
	Yes	148	40.30%
	I don’t know	184	50.10%
Are imaging tests such as ultrasound or MRI always necessary to diagnose lipomas?	No	28	7.60%
	Yes	164	44.70%
	I don’t know	175	47.70%
Is treatment usually required for lipomas?	No	68	18.50%
	Yes	136	37.10%
	I don’t know	163	44.40%
Can larger or symptomatic lipomas be treated differently than smaller ones?	No	18	4.90%
	Yes	176	48.00%
	I don't know	173	47.10%
Is it common to only monitor the growth of a lipoma before recommending treatment?	No	36	9.80%
	Yes	143	39.00%
	I don’t know	188	51.20%
Is surgery the only method for removing lipomas?	No	80	21.80%
	Yes	95	25.90%
	I don’t know	192	52.30%
Is there a risk of lipoma recurrence after surgical removal?	No	22	6.00%
	Yes	140	38.10%
	I don’t know	205	55.90%
On the scale of 1 to 5, how well are you aware of the simple surgical excision of lipomas?	1	181	49.30%
	2	67	18.30%
	3	70	19.10%
	4	16	4.40%
	5	33	9.00%

Personal experiences and barriers to seeking medical intervention regarding lipoma

The study examined the limitations and barriers hindering individuals from seeking medical attention for lipoma presentations, with the majority citing unawareness and lack of knowledge about lipomas and surgical excision (N = 201, 54.80%) as a significant obstacle. Regarding personal experiences with lipomas, a vast majority (N = 314, 85.60%) reported never being diagnosed with one, while a small percentage (N = 15, 4.10%) had been diagnosed, predominantly with single lipomas (N = 22, 6.00%). The most common site of lipomas reported among those diagnosed was the upper extremity (N = 17, 4.60%). Pain or discomfort (N = 32, 8.70%) was the most commonly mentioned characteristic of lipoma. Furthermore, 46.00% (N = 169) of participants did not know if there were known cases of lipoma among close relatives. Among those with diagnosed lipomas, only a fraction sought medical attention (N = 15, 4.10%), with reasons for not seeking treatment including fear of diagnosis or surgery (N = 24, 10.40%). Simple excision procedures were uncommon (N = 10, 2.70%), and recurrence rates were low (N = 9, 2.50%). However, among those who experienced recurrence, new lipomas were commonly located in different areas (N = 10, 3.20%) (Table 4).

**Table 4:** Personal experiences and barriers to seeking medical intervention regarding lipoma (n = 367). Data are expressed as n and %.

Parameter	Category	N	%
In your opinion, what are the limitations and barriers that hinder people from seeking medical attention for lipoma presentation in some cases?	Cosmetic concerns	88	24.00%
	Fear of medical procedures	165	45.00%
	Risk of complications	98	26.70%
	Risk of recurrence	90	24.50%
	Difficulty in the reach of medical centers (rural areas, cost, transportation, etc.)	83	22.60%
	Unawareness and lack of knowledge about lipomas and surgical excision	201	54.80%
	Other	56	15.30%
Have you ever been diagnosed with lipoma?	No	314	85.60%
	Yes	15	4.10%
	I don’t know	38	10.40%
Was it single or multiple?	Single	22	6.00%
	Multiple	13	3.50%
	I have never had a lipoma before	332	90.50%
Where was the lipoma?	Upper extremity (hands, arms, etc.)	17	4.60%
	Trunk	11	3.00%
	Lower extremities (hips, legs, and feet)	5	1.40%
	Other	8	2.20%
	I have never had a lipoma before	326	88.80%
What were the characteristics of the lipoma?	Painless	24	6.50%
	Firm and hard	11	3.00%
	Soft or moving under the skin	20	5.40%
	Pain or discomfort	32	8.70%
	Other	12	3.30%
	I have never had a lipoma before	328	89.40%
Are there any known cases of lipoma among your close relatives?	No	128	34.90%
	Yes	70	19.10%
	I don’t know	169	46.00%
Did you seek any medical attention?	No	69	18.80%
	Yes	15	4.10%
	I have never had a lipoma before	283	77.10%
If the answer is no, why?	It was small and asymptomatic	21	9.10%
	I wasn’t aware of the nature of it or the simple excision procedure	23	10.00%
	Fear of diagnosis or surgery	24	10.40%
	Other	162	70.40%
Did you undergo a simple excision procedure for the lipoma?	No	57	15.50%
	Yes	10	2.70%
	I have never had a lipoma before	300	81.70%
Have you developed any additional lipomas after undergoing simple excision?	No	42	11.40%
	Yes	9	2.50%
	I have never had a lipoma before	316	86.10%
If yes, where are they located?	In the same place as the previous one	5	1.60%
	In another location	10	3.20%
	I have never had a lipoma before	293	95.10%

The association of sociodemographic data with the knowledge level of lipoma

The analysis of demographic factors and their association with median levels of awareness about lipomas and simple surgical excision revealed some notable trends. There were significant differences concerning marital status (p = 0.019). Specifically, individuals who were widowed exhibited the highest median awareness level, with a median score of 9.0 (IQR = 2.0-10.0), followed by single participants with a median score of 8.0 (IQR = 2.0-11.0). Age, gender, educational level, employment status, and residence did not show significant differences in median awareness levels (p > 0.05). These findings suggest that marital status may influence awareness levels about lipomas and simple surgical excision, warranting further investigation (Table 5).

**Table 5:** Association of different sociodemographic variables with the knowledge level of lipoma. Data are expressed as median (IQR). *: p < 0.05. IQR = interquartile range

Parameter	Category	Median (IQR)	P-value
Age (year)	18–29	8.0 (1.0–11.0)	0.070
	30–39	6.0 (0.0–12.25)	
	40–49	4.0 (0.25–9.0)	
	50–59	7.0 (1.0–11.0)	
	60 or more	2.0 (0.0–8.5)	
Gender	Female	7.0 (1.0–10.0)	0.446
	Male	6.0 (0.0–11.0)	
Marital status	Single	8.0 (2.0–11.0)	0.019*
	Married	5.0 (0.0–10.0)	
	Divorced	5.0 (0.0–10.0)	
	Widowed	9.0 (2.0–10.0)	
	Other	0.0 (0.0–4.5)	
Educational level	Primary	4.0 (0.0–not mentioned)	0.153
	Middle	0.0 (0.0–not mentioned)	
	High	7.5 (1.0–11.0)	
	Diploma	5.5 (0.0–9.0)	
	Bachelor's degree	6.5 (1.0–10.0)	
	Other	10.0 (5.5–12.5)	
Employment status	Student	8.0 (2.5–11.0)	0.060
	Employed	6.0 (0.0–10.0)	
	Not employed	5.5 (0.0–10.0)	
	Retired	5.0 (0.0–11.0)	
	Other	4.5 (1.0–10.0)	
Residence	Qunfudhah	6.0 (0.0–10.0)	0.333
	The Holy Capital	6.0 (0.25–11.0)	
	Jeddah	7.0 (4.0–10.0)	
	Rabigh	12.0 (10.0–not mentioned)	
	Taif	5.5 (0.75–11.0)	
	Leith	8.0 (1.5–11.5)	
	Other	6.5 (1.0-10.0)	

The association of different sociodemographic variables with risk factors for lipoma

The analysis of demographic factors and their association with median levels of risk factors for lipoma revealed several significant findings. Age was found to have a significant impact on satisfaction levels (p = 0.019), with individuals aged 18-29 years exhibiting the highest median score of 2.0 (IQR = 0.0-3.0). Marital status also showed significant differences (p = 0.014), with single individuals reporting the highest median level of 2.0 (IQR = 0.0-3.0). Employment status demonstrated significant differences (p = 0.006), with students reporting the highest median level of 2.0 (IQR = 0.0-3.0). No significant differences were observed based on gender, educational level, or residence (Table 6).

**Table 6:** Association of different sociodemographic variables with risk factors for lipoma. Data are expressed as median (IQR). *: p < 0.05. IQR = interquartile range

Parameter	Category	Median (IQR)	P-value
Age (year)	18–29	2.0 (0.0–3.0)	0.019*
	30–39	0.5 (0.0–4.0)	
	40–49	1.0 (0.0–2.0)	
	50–59	1.0 (0.0–3.0)	
	60 or more	0.0 (0.0–1.5)	
Gender	Female	1.0 (0.0–3.0)	0.363
	Male	1.0 (0.0–3.0)	
Marital status	Single	2.0 (0.0–3.0)	0.014*
	Married	1.0 (0.0–3.0)	
	Divorced	0.0 (0.0–3.5)	
	Widowed	1.0 (0.0–2.5)	
	Other	0.0 (0.0–1.0)	
Educational level	Primary	0.0 (0.0–not mentioned)	0.150
	Middle	0.0 (0.0–not mentioned)	
	High	1.5 (0.0–3.0)	
	Diploma	0.5 (0.0–2.0)	
	Bachelor's degree	1.0 (0.0–3.0)	
	Other	2.0 (0.0–4.0)	
Employment status	Student	2.0 (0.0–3.0)	0.006*
	Employed	1.0 (0.0–3.0)	
	Not employed	1.0 (0.0–2.25)	
	Retired	1.0 (0.0–3.0)	
	Other	1.0 (0.0–2.5)	
Residence	Qunfudhah	1.0 (0.0–3.0)	0.346
	The Holy Capital	1.0 (0.0–3.0)	
	Jeddah	2.0 (0.0–3.0)	
	Rabigh	4.0 (1.0–not mentioned)	
	Taif	1.0 (0.0–3.0)	
	Leith	2.0 (0.0–4.75)	
	Other	1.5 (0.0–2.25)	

The analysis examining the association between demographic factors and risk factors for lipoma revealed several noteworthy findings. Age groups ranging from 18-29 to 50-59 years demonstrated positive beta coefficients, indicating a positive association with risk factors for lipoma, although none reached statistical significance (p > 0.05). Similarly, across marital status categories, including single, married, divorced, and widowed, positive beta coefficients were observed, suggesting a potential positive association with risk factors for lipoma, albeit without statistical significance (p > 0.05). Employment status categories, including student, employed, not employed, and retired, showed mixed results, with some categories displaying negative beta coefficients, indicating a negative association with risk factors for lipoma, but none of these associations were statistically significant (p > 0.05). These findings suggest that while there may be trends indicating associations between demographic factors and satisfaction levels, further investigation with larger sample sizes may be necessary to establish significant relationships. The multivariable generalized linear regression analysis assessed the independent predictors of knowledge regarding lipomas using the significantly associated variables as independent variables, and the dependent variable was the percent knowledge score. Results were presented as beta coefficients and 95% confidence intervals. Statistical significance was set at p-values <0.05 (Table 7).

**Table 7:** Linear regression showing the relationship of the statistically significant sociodemographic variables with risk factors for lipoma. CI = confidence interval; LB = lower bound; UB = upper bound

Parameter	Category	Beta	95% CI		P-value
			LB	UB	
Age (year)	18–29	1.170	-0.859	3.199	0.258
	30–39	1.641	-0.394	3.675	0.114
	40–49	0.947	-1.025	2.918	0.346
	50–59	1.192	-0.734	3.118	0.224
	60 or more	Ref.	Ref.	Ref.	Ref.
Marital status	Single	1.411	-0.174	2.997	0.081
	Married	1.339	-0.270	2.948	0.103
	Divorced	1.499	-0.333	3.332	0.108
	Widowed	1.427	-0.569	3.423	0.161
	Other	Ref.	Ref.	Ref.	Ref.
Employment status	Student	0.438	-0.645	1.520	0.427
	Employed	-0.132	-1.117	0.853	0.792
	Not employed	-0.297	-1.364	0.770	0.585
	Retired	0.052	-1.330	1.433	0.942
	Other	Ref.	Ref.	Ref.	Ref.

## Discussion

This study aimed to evaluate lipoma awareness and surgical excision knowledge among residents in the Makkah region. The results provided insights into lipoma from different aspects, including sociodemographic data, general knowledge about lipomas, and their risk factors, diagnosis, and medical management related to these soft tissue tumors.

Additionally, the study investigated the respondents’ personal experiences and the barriers to not seeking medical attention. It showed the association of sociodemographic data with knowledge and risk factors of lipomas.

In terms of sociodemographic data, the majority of the participants were 18-29 years old (56.10%), predominantly female (74.10%), and mostly held a bachelor’s degree (59.40%), and a large proportion of the respondents were students (40.60%). Therefore, these findings highlighted that people with higher educational backgrounds were well represented, which indicates that education could be a fundamental solution to improve awareness and knowledge on various topics.

The study showed that while 42.80% of respondents were ignorant of lipomas, 48.50% had heard of them. However, there was uncertainty and misconceptions regarding various aspects, such as growth characteristics, risk factors, associated pain and symptoms, and management options. Therefore, it highlights the need to correct these misconceptions to improve public understanding.

Of the participants, 45.50% had no awareness of lipomas, whereas 26.70% of the respondents obtained their information about lipomas via social media platforms, highlighting the importance of social media platforms in improving health-related knowledge. Regarding management, 25.90% of participants thought that surgery was the only way to remove the tumor, and 38.10% were aware that there was a chance that lipoma may return after surgery.

Badhiya et al. in a study published in 2022 found no relationship between the rate of lipoma recurrence and the patient’s age, sex, or size and site of lipoma [8]. Although the marginal excision of lipoma has been a standard, Kooby et al. reported a higher risk of local recurrence after the marginal excision of lipomas [9]. In another study, Sommerville et al. reported a local recurrence rate of 8% after the marginal excision of lipoma [10], similar to the study by Bassett et al. which also recommended the marginal excision of lipoma because of the low risk of local recurrence of lipomas [11].

Furthermore, 49.30% of participants reported having the lowest level of awareness about simple surgical excision. Regarding personal experiences with lipomas, a vast majority (85.60%) reported never being diagnosed with one, while a small percentage (4.10%) had been diagnosed, predominantly with single lipomas (6.00%). The upper extremity (4.60%) was the most often reported site of lipomas among the diagnosed individuals. Merely 4.10% of individuals with lipomas sought medical attention, and 10.40% of the respondents cited fear of the diagnosis or surgery as their reason for not seeking treatment. Simple excision procedure was rare (2.70%).

One study discussed seeking medical attention for lipomas. The study identified a crucial factor associated with the clinical incidence of seeking medical care. Hence, many people with lipomas might not have had their diagnosis noted when they saw their doctors for other conditions. The study then showed that the true number of lipomas may be more than the number recorded in medical records and may even be roughly twice as high as the number of lipomas that were microscopic examined for histological analysis [12].

In a study by Ezike et al., the most common site for lipomas was the head and neck, followed by the trunk, accounting for 75.52% of cases. Additionally, 30.15% of lipomas affected the forearm [13]. In our study, respondents reported experiencing lipomas most commonly in the upper extremities, followed by the trunk. Moreover, multiple subcutaneous lipomas were reported in 22 (17.46%) patients. However, lipomas can also appear in significantly rare sites, as in a case of lingual lipoma reported from Makkah in 2020.

The first reported case of lipoma of the tongue was from Saudi Arabia [14]. Another case was reported in Jeddah in 2022 of a vulvar lipoma in a 29-year-old female, which was removed by surgical excision [15].

Regarding the association between sociodemographic data and the knowledge level of lipoma, there were significant differences related to marital status (p = 0.019). Specifically, respondents who were widowed demonstrated the highest median awareness level, followed by single participants. These results suggest that marital status potentially impacts awareness levels about lipomas and simple surgical excision, indicating the need for additional investigation.

Although this research provides valuable insights into lipomas, it is important to address certain limitations of our study, such as the sparse literature on lipoma awareness due to the limited number of previous studies on this topic. Our study is a cross-sectional online survey that limits our ability to examine cause-effect relationships, which may have restricted the perception of illiterate individuals. Additionally, the study was restricted to residents of the Makkah region, with the majority of participants recruited from Al-Qunfudhah and the Holy Capital. Third, the sample size was relatively limited. Furthermore, results may be skewed due to the large proportion of people who have never had lipoma.

## Conclusions

The study aimed to assess the awareness of lipoma and surgical excision knowledge among residents from the Makkah region. The study findings indicated a moderate level of awareness of lipoma (48.5%). However, 31.1% were dissatisfied with the lipoma information that is currently available, and there was a lack of knowledge regarding surgical excision of lipoma and the risk of recurrence. There is still scope for improvement in terms of knowledge on this topic. Healthcare professionals play the most significant role in informing the public about lipoma and how it differs from other skin conditions. These efforts could help address misconceptions, improve treatment decision-making, and, ultimately, contribute to better health outcomes for individuals affected by lipomas.

## Appendices

Questionnaire

1- Are imaging tests like ultrasound and MRI always necessary to diagnose lipomas?

- Yes

- No

- I do not know

Seventh section: Knowledge about medical intervention.

1- Is treatment usually required for lipomas?

- Yes

- No

- I do not know

2- Is surgery the only method for removing lipomas?

- Yes

- No

- I do not know

3- Can larger or symptomatic lipomas be treated differently than smaller ones?

- Yes

- No

- I do not know

4- Is it common to only monitor the growth of a lipoma before recommending treatment?

- Yes

- No

- I do not know

5- Is there a risk for lipoma recurrence after surgical removal?

- Yes

- No

- I do not know

6- On a scale of 1 to 5, how well are you aware of the simple surgical excision of lipomas?

- 1

- 2

- 3

- 4

- 5

Eighth section: Barriers to not seeking medical attention.

1- In your opinion, what are the limitations and barriers that prevent patients from seeking medical attention for lipoma presentation in some cases?

- Cosmetic concerns

- Fear of medical procedures

- Risk of complications

- Risk of recurrence

- Difficulty in the reach of medical centers ( rural areas, cost, transportation, etc.)

- Unawareness and lack of knowledge about lipomas and surgical excision

- Other?

Ninth section: Personal experiences.

1- Have you ever been diagnosed with lipoma?

- Yes

- No

- I have never had a lipoma before

2- Was it single or multiple?

- Single

- Multiple

- I have never had a lipoma before

3- Site of the lipoma?

- Upper extremity (hand, arms, etc.)

- Trunk

- Lower extremity (hip, legs, feet)

- Other

- I have never had a lipoma before

4- What were the characteristics of lipoma?

- Painless

- Firm and hard

- Soft and moving under the skin

- Pain or discomfort

- Slow growing

- Other?

- I have never had a lipoma before

5- Are there any known cases of lipoma among your close relatives?

- Yes

- No

- I do not know

6- Do you seek any medical attention?

- Yes

- No

- I do not know

7- If the answer is no, why?

- It was small and asymptomatic

- I was not aware of the nature of it and the simple excision procedure

- Fear of diagnosis or surgery

- Other?

8- Did you undergo a simple excision procedure for the lipoma?

- Yes

- No

- I have never had a lipoma before

9- Have you ever developed any additional lipomas after undergoing the simple excision?

- Yes

- No

- I have never had a lipoma before

10- If yes, where are they located?

- In the same place as the previous one.

- In another location

- I have never had a lipoma before
